# Inhibition of PTEN activates bovine non-growing follicles *in vitro* but increases DNA damage and reduces DNA repair response

**DOI:** 10.1093/humrep/dey354

**Published:** 2018-12-06

**Authors:** Mila Maidarti, Yvonne L Clarkson, Marie McLaughlin, Richard A Anderson, Evelyn E Telfer

**Affiliations:** 1 MRC Centre for Reproductive Health, Queens Medical Research Institute, University of Edinburgh, Edinburgh, UK; 2 Institute of Cell Biology and Genes and Development Group, CDBS Hugh Robson Building, University of Edinburgh, Edinburgh, UK

**Keywords:** PTEN inhibition, non-growing follicle activation, DNA damage, DNA repair, bovine ovarian follicles, *in vitro*

## Abstract

**STUDY QUESTION:**

Does ovarian follicle activation by phosphatase homologue of chromosome-10 (PTEN) inhibition affect DNA damage and repair in bovine oocytes and granulosa cells?

**SUMMARY ANSWER:**

PTEN inhibition promotes bovine non-growing follicle activation but results in increased DNA damage and impaired DNA repair capacity in ovarian follicles *in vitro*.

**WHAT IS KNOWN ALREADY:**

Inhibition of PTEN is known to activate primordial follicles but may compromise further developmental potential. In breast cancer cells, PTEN inhibition represses nuclear translocation of breast cancer susceptibility 1 (BRCA1) and Rad51; this impairs DNA repair resulting in an accumulation of damaged DNA, which contributes to cell senescence.

**STUDY DESIGN, SIZE, DURATION:**

Bovine ovarian tissue fragments were exposed to control medium alone or containing either 1 or 10 μM bpv(HOpic), a pharmacological inhibitor of PTEN, *in vitro* for 24 h. A sub-group of tissue fragments were collected for Western blot analysis after bpv(HOpic) exposure. The remainder were incubated in control medium for a further 5 days and then analysed histologically and by immunohistochemistry to detect DNA damage and repair pathways.

**PARTICIPANTS/MATERIALS, SETTING, METHODS:**

Bovine ovaries were obtained from abattoir-slaughtered heifers. Tissue fragments were exposed to either control medium alone or medium containing either 1 μM or 10 μM bpv(HOpic) for 24 h. Tissue fragments collected after 24 h were subjected to Akt quantification by Western blotting (six to nine fragments per group per experiment). Follicle stage and morphology were classified in remaining fragments. Immunohistochemical analysis included nuclear exclusion of FOXO3 as a marker of follicle activation, γH2AX as a marker of DNA damage, meiotic recombination 11 (MRE11), ataxia telangiectasia mutated (ATM), Rad51, breast cancer susceptibility 1 (BRCA1) and breast cancer susceptibility 2 (BRCA2) as DNA repair factors. A total of 29 550 follicles from three independent experiments were analysed.

**MAIN RESULTS AND THE ROLE OF CHANCE:**

Tissue fragments exposed to bpv(HOpic) had increased Akt phosphorylation at serine 473 (pAkt/Akt ratio, 2.25- and 6.23-fold higher in 1 and 10 μM bpv(HOpic) respectively compared to control, *P* < 0.05). These tissue fragments contained a significantly higher proportion of growing follicles compared to control (78.6% in 1 μM and 88.7% in 10 μM versus 70.5% in control; *P* < 0.001). The proportion of morphologically healthy follicles did not differ significantly between 1 μM bpv(HOpic) and control (*P* < 0.001) but follicle health was lower in 10 μM compared to 1 μM and control in all follicle types (*P* < 0.05). DNA damage in oocytes, indicated by expression of γH2AX, increased following exposure to 1 μM bpv(HOpic) (non-growing, 83%; primary follicles, 76%) and 10 μM (non-growing, 77%; primary, 84%) compared to control (non-growing, 30% and primary, 59%) (*P* < 0.05 for all groups). A significant reduction in expression of DNA repair proteins MRE11, ATM and Rad51 was observed in oocytes of non-growing and primary follicles of treatment groups (primary follicles in controls versus 10 μM bpv(HOpic): MRE, 68% versus 47%; ATM, 47% versus 18%; Rad51, 48% versus 24%), *P* < 0.05 for all groups. Higher dose bpv(HOpic) also resulted in lower expression of BRCA1 compared to control and 1 μM bpv(HOpic) (*P* < 0.001) in non-growing and primary follicles. BRCA2 expression was increased in oocytes of primary follicles in 1 μM bpv(HOpic) (36%) compared to control (20%, *P* = 0.010) with a marked decrease in 10 μM (1%, *P* ≤ 0.001). Granulosa cells of primary and secondary follicles in bpv(HOpic) groups showed more DNA damage compared to control (*P* < 0.05). However, bpv(HOpic) did not impact granulosa cell DNA repair capacity in secondary follicles, but BRCA1 declined significantly in higher dose bpv(HOpic).

**LARGE-SCALE DATA:**

N/A.

**LIMITATIONS, REASONS FOR CAUTION:**

This study focuses on non-growing follicle activation after 6 days culture and may not reflect DNA damage and repair capacity in later stages of oocyte and follicle growth.

**WIDER IMPLICATIONS OF THE FINDINGS:**

*In vitro* activation of follicle growth may compromise the bidirectional signalling between oocyte and granulosa cells necessary for optimal oocyte and follicle health. This large animal model may be useful in optimising follicle activation protocols with a view to transfer for clinical application.

**STUDY FUNDING/COMPETING INTEREST(S):**

This work was supported by Indonesia endowment fund for education. No competing interest.

**TRIAL REGISTRATION NUMBER:**

Not applicable.

## Introduction

The phosphatidylinositol 3-kinase (PI3K) signalling pathway appears to be the primary non-gonadotrophic growth factor signalling pathway that regulates the growth and differentiation of ovarian follicles (Dupont and Scaramuzzi, 2016). The balance between PI3K/Akt substrates determines follicle growth acceleration, deceleration, survival and apoptosis (Liu *et al.*, 2006; Zhou *et al.*, 2017), and phosphatase homologue of chromosome-10 (PTEN) is a negative regulator of this pathway. Excessive PI3K activation in mice has been hypothesised to contribute to premature activation of primordial follicles which in turn results in depletion of the primordial follicle pool and ovarian aging (Reddy *et al.*, 2008; Sobinoff *et al.*, 2012). Inhibition of PTEN in cultured human ovarian cortex results in increased activation of primordial follicles and more secondary follicles, however the subsequent growth and survival of those apparently healthy isolated secondary follicles is compromised (McLaughlin *et al.*, 2014; Grosbois and Demeestere, 2018).

This finding might be related to the role of PTEN in maintaining genomic integrity (Shen *et al.*, 2007), promoting and regulating cell growth and survival (Reddy *et al.*, 2008; Jagarlamudi *et al.*, 2009). Akt activation during cell cycles in normal cell proliferation upregulates numerous substrates at the G1/S and G2/M transition, some of which are involved in DNA damage repair pathway. DNA damage is the starting event of apoptosis and can be detected even in the absence of morphological changes. It is suggested that the PI3K/Akt pathway initiates checkpoint kinase 1 (Chk1) phosphorylation during DNA damage response cascade at G2 arrest (Xu *et al.*, 2010), thus allowing time for DNA repair processing.

Effects of PTEN inhibition on DNA damage response have been reported in many different types of cancer (Altiok *et al.*, 1999; Plo *et al.*, 2008; Golding *et al.*, 2009; Fraser *et al.*, 2012) with varying outcomes. Endogenously high levels of Akt decreases homologous recombination repair capacity of DNA double-strand breaks (DSBs) (Brunet *et al.*, 1999; Thacker, 2005; Plo *et al.*, 2008; Jia *et al.*, 2013). In addition, a study using a breast cancer cell line showed that high intracellular levels of Akt repressed nuclear translocation of breast cancer susceptibility 1 (BRCA1) and Rad51, resulting in the lack of homologous recombination of DNA DSB repair (Plo *et al.*, 2008). Upregulation of the PI3K/Akt pathway can also generate spontaneous DNA breaks and pose a significant threat to genome stability by inhibition of Chk1 (Puc and Parsons, 2005). On the other hand, low protein kinase B (Akt) activity has been shown to impair the DNA damage repair mechanism by non-homologous end joining in human glioma cells (Kao *et al.*, 2007; Golding *et al.*, 2009).

Taken together, these findings support the idea that oocytes lacking PTEN may accumulate DNA damage, with reduced DNA damage repair capacity. DNA DSBs are the most detrimental type of damage, but they do not occur as frequently as other lesions. Persistent unrepaired DNA DSBs may lead to genomic instability (Khanna and Jackson, 2001; Jackson and Bartek, 2009; Menezo *et al.*, 2010; Titus *et al.*, 2013) and the capacity of the cell to repair the damage will influence the balance between cell survival and apoptosis (Bzymek *et al.*, 2010; Torgovnick and Schumacher, 2015). In oocytes and granulosa cells, unrepaired DNA DSBs may potentially impact upon the quality of oocytes (Carroll and Marangos, 2013; Oktay *et al.*, 2015; Winship *et al.*, 2018). In this study, our aim was to determine whether PTEN inhibition affected DNA damage and repair mechanisms in bovine ovarian follicles activated *in vitro*, using a serum-free culture system. We have shown that this system is able to maintain follicular growth and support oocyte development *in vitro* using bovine and human ovaries (Telfer *et al.*, 2008; McLaughlin and Telfer, 2010).

## Materials and Methods

### Ovarian cortical tissue collection, preparation

Bovine ovaries were obtained from the abattoir and collected in pre-warmed culture medium M199 (HEPES buffered) (Gibco BRL, Life Technologies Ltd., Paisley, Renfrewshire, UK) supplemented with sodium pyruvate (2 mM), glutamine (2 mM), bovine serum albumin (BSA) (3 mg/ml), penicillin G (75 μg/ml), streptomycin (50 μg/ml) and amphotericin B (2.5 μg/ml) (all chemicals from Sigma-Aldrich Chemicals, Poole, Dorset, UK). At the laboratory, thin slices of ovarian cortex were removed from the ovaries using a scalpel blade no. 24 and then transferred into fresh dissection medium comprising preheated Leibovitz medium (Gibco BRL) supplemented with sodium pyruvate (2 mM), glutamine (2 mM), BSA (Fraction V. 3 mg/ml), penicillin G (75 μg/ml) and streptomycin (50 μg/ml). Excess stromal tissue was trimmed using forceps and a scalpel blade. The tissue was gently stretched using the blunt edge of a scalpel blade with the cortex uppermost and cut into small strips sized 4 mm × 2 mm × 1 mm. Any follicles measuring >40 μm were excised from the tissue fragments.

### Ovarian tissue fragments culture

Basic culture medium was prepared from McCoy’s 5a medium with bicarbonate and HEPES (20 mM) (GIbco BRL) supplemented with BSA (1 mg/ml), glutamine (3 mM), penicillin G (0.1 mg/ml), streptomycin (0.1 mg/ml), transferrin (2.5 μg/ml), sodium selenite (4 ng/ml), insulin (l0 ng/ml), hFSH (1 ng/ml) and ascorbic acid (50 μg/ml) (all obtained from Sigma-Aldrich Chemicals). Before use, the medium was equilibrated at 37°C in humidified air with 5% CO_2_.

Following tissue preparation and cutting, 10–12 fragments per culture were randomly selected as 0 h controls for histological examination. The remaining tissue fragments were cultured in flat-bottomed 24-well culture plates (Corning Costar Europe, Badhoevedorp, The Netherlands) containing 300 μl of basic culture medium or culture medium supplemented with the PTEN inhibitor dipotassium bisperoxo(5-hydroxypyridine-2-carboxyl) oxovanadate (V) (bpv(HOpic) (Merck Millipore Chemicals Ltd, UK) at 1 or 10 μM at 37°C in humidified air with 5% CO_2_. After 24 h, all media was removed from tissue fragments and replaced with fresh basic culture medium. At this point, 6–9 tissue fragments from each group were snap-frozen and stored at −80°C for Western blot analysis of Akt phosphorylation.

Remaining tissue fragments were incubated for a further 5 days, with half the media removed and replaced with fresh on alternate days. On completion of the culture period, all remaining tissue fragments were fixed in 10% normal buffered formalin (NBF) for histological examination to determine the effect of PTEN inhibitor on follicle and oocyte development.

### Histological methods and tissue analysis

After fixation in NBF for 24 h, tissues were further processed and embedded individually into paraffin wax blocks and serially sectioned at 5 μm thickness. Sections were mounted on Super Frost Plus slides (VWR International Ltd., Leicestershire, UK). For all morphological and numerical analyses, the examiner was blinded to the treatment groups. Analysis of follicles within tissue fragments was performed on every section under the light microscope with a crossed micrometre under 40× magnification. Follicle developmental stage was categorised using a modification of an established system (Pedersen and Peters, 1968). Primordial and transitory follicles were classified as non-growing due to the evidence suggesting that in the bovine ovary these follicles are in quiescence (van Wezel and Rodgers, 1996). The number of follicles within each stage of development was recorded, for Day 0 and Day 6 of each treatment. The classification of healthy follicles was based on the same criteria as in Telfer *et al.* with modifications (Telfer *et al.*, 2008). For follicles to be categorised as morphologically normal the oocyte must be grossly circular, surrounded by a zona pellucida, have a visible germinal vesicle and defined nucleolus and have <10% of pyknotic granulosa cells present. The proportion of follicles at different developmental stages was defined as a percentage of morphologically healthy follicles over the total follicle count (Brunet *et al.*, 1999).

### Immunohistochemistry

Quantitative analysis of DNA damage was performed using immunofluorescence. DNA damage repair proteins were localised in tissue sections using antibodies against Rad51 (137323; 1:500; Abcam), meiotic recombination (MRE) 11 (NB100-142; 1:1000; Novusbio), BRCA1 (Ab16781; 1:200; Abcam), ATM (ab78; 1:500; Abcam) and BRCA2 (Ab27976; 1:200; Abcam). Nuclear exclusion of FOXO3 was detected using immunohistochemistry (NBP2-24579; 1:500; Novusbio).

Tissue sections mounted on slides were dewaxed in xylene and rehydrated through decreasing concentrations of alcohol before being immersed in tris-buffered saline with 0.05% (v/v) Tween 20 (TBST). Antigen retrieval was performed by microwaving the slides in 10 mM sodium citrate (pH 6.0) at simmer setting for 20 min. Following antigen retrieval, the slides were washed in TBST (2 × 5 min) and then immersed in 3% (v/v) hydrogen peroxide to quench endogenous peroxidase activity. After 2 × 5 min washes in TBST, sections were incubated with appropriate blocking solution for 1 h (150 μl goat serum or horse serum in 10 ml TBST followed by overnight incubation with the diluted primary antibodies at 4°C. Primary antibody was replaced with blocking solution for negative controls.

Primary antibody was washed off, and sections were incubated for 30 min with biotinylated secondary antibody at room temperature (Vectastain Elite ABC kit; Vector Laboratories, Peterborough, UK) and then washed in TBST (2 × 5 min). Slides were then incubated with Streptavidin Horseradish Peroxidase (Streptavidin horse-radish peroxidase; HRP) for 30 min at room temperature. Following a TBST wash DAB (3,3′-diaminobenzidine) peroxidase substrate kit (Vector Laboratories) solution was added to the sections for between 2 and 5 min and counterstained with haematoxylin for 20 s, dehydrated in graded alcohol, cleared and then mounted with dibutylphthalate polystyrene xylene (DPX).

### Immunofluorescence

Localisation of γH2AX (a marker of DNA damage) was detected by immunofluorescence. As previously described mounted tissue sections were deparaffinised and rehydrated and then washed in PBS with 0.1% (v/v) Triton X-100 (PBST) (pH 7.2–7.4) for 2 × 5 min. Then, the slides were subjected to high temperature antigen retrieval as described earlier and incubated for 1 h at RT with blocking solution (5%, v/v, goat serum in PBST). Tissue sections were then probed with primary antibody (1:1000) against γH2AX (NB100-384; Novusbio) overnight at 4°C. Blocking solution without primary antibody served as negative control. After washing with PBST (4 × 10 min), sections were incubated with appropriate secondary antibodies (Cy3-conjugated affinity pure donkey anti rabbit IgG [H + L], 1:250; Jackson Laboratories) for 2 hours and then washed for 2 × 10 min. The slides were then mounted in Vectashield hardset with 4′-6-diamidino-2-phenylindole (DAPI) (H-1500; Vector Laboratories).

Images were analysed using ImageJ and γH2AX expression in oocytes and granulosa cells determined. The number of oocytes with γH2AX foci per total number of follicles was calculated. The γH2AX expression in granulosa cells was quantitatively analysed by calculating the proportion of γH2AX positive granulosa cells per total number of granulosa cells per follicle. Images were captured using a Zeiss LSM 800 confocal microscope with X20 magnification in the IMPACT imaging facility (Centre for Discovery Brain Sciences, The University of Edinburgh).

### Western blotting

Ovarian cortical strips (6–9 per group per experiment) were suspended in radio immunoprecipitation assay buffer (RIPA) extraction buffer (Fisher Scientific, Loughborough, UK) supplemented with 1% v/v Halt Protease and Phosphatase Inhibitor Cocktail (PI) (Thermo Scientific, Loughborough, UK). The tissue was cut with scissors and homogenised. Proteins were detected using a slightly modified protocol as previously described (Clarkson *et al.*, 2018). In brief, the sample was centrifuged at 3400 × *g* for 5 min and protein was purified using Vivaspin tubes (Sartorius Mechatronics Ltd, Epsom, UK) with 50 kDa filters. Protein concentration was measured using Coomassie-Plus Reagent (Thermo Scientific Pierce, Northumberland, UK). Protein samples were denatured at 100°C for 10 min, 20 μg was loaded onto 4–20% gradient gels (Life Technologies, Paisley, UK) in Tris-glycine/SDS running buffer (25 mM Tris-HCl, 52 mM glycine, 0.1% SDS) and run at 125 V for 1 h. Proteins were transferred to nitrocellulose membranes (Amersham Pharmacia). Bovine serum albumin (BSA) (5%, w/v) in TBST was used to block the nitrocellulose membranes for 1 h at room temperature with gentle agitation. Blots were then incubated, with a rabbit monoclonal antibody against Akt (9272, 1:5000; Cell Signaling) or rabbit polyclonal antibody against Akt phosphorylated at serine 473 (ab81283, 1:500; Abcam) and with a mouse monoclonal antibody against alpha tubulin (ab7291, 1:5000; Abcam) as a loading control, overnight at 4°C with gentle agitation. Blots were washed in 0.1% TBST and then incubated with appropriate secondary antibodies, a goat polyclonal antibody raised against mouse IgG (heavy and light chain) (115-035-146; Jackson Laboratory) or rabbit IgG (H + L) (111-035-003) 1:5000 in 5% BSA for 1 h at room temperature. To enhance chemiluminescence detection, nitrocellulose membranes were placed in Amersham (ECL) prime Western blotting detection reagent (GE Health Care) for 1 min and exposed to autoradiographic film. Western blots were digitally scanned and analysed using ImageJ. All analysis was normalised to alpha tubulin.

### Statistical analyses

All data were analysed using the SPSS statistical software version 22 (SPSS, Inc., Chicago, USA) and GraphPad Software version 7 (GraphPad Software Inc., San Diego, CA, USA). Quantitative data are presented as mean ± SEM. Chi-squared test was used to analyse the percentage of healthy and unhealthy follicles, the distribution of follicle stages and the proportion of oocytes expressing proteins related to DNA damage and DNA DSB repair. Granulosa cell expression of γH2AX, MRE11, ATM, Rad51, BRCA1 and BRCA2 was determined using one-way ANOVA test followed by Bonferroni *post hoc* test. Statistical significance was assigned at *P* ≤ 0.05.

## Results

### Analysis of follicle distribution

**Figure 1:**
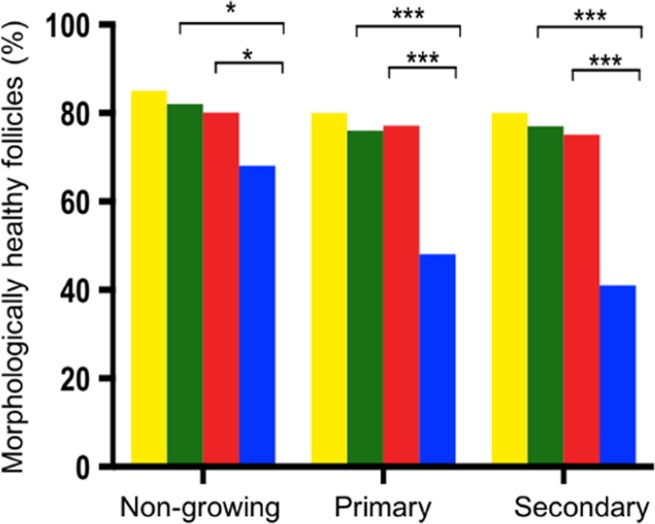
**Proportion of morphologically healthy follicles at each stage of development**. Day 0 (yellow), control medium (green), 1 μM bpv(HOpic) (red) and 10 μM bpv(HOpic) (blue). ***≤0.001, **≤0.01 and *≤0.05. The total number of follicles analysed for each stage and treatment is shown in Table I. Data here represent the proportion that were classified as healthy.

A total of 32 ovarian cortical fragments were obtained on Day 0 from three culture replicates, and a total of 8833 follicles were analysed. Non-growing follicles were the most prevalent on Day 0, constituting 79.6% of total follicle number. The majority of the remaining follicles were at the primary stage (19.0%) and a small percentage were at secondary stage (1.4%) (Table I). More than 80% of all follicles at Day 0 were healthy (Fig. 1). No antral follicles were observed at Day 0 (D0) in any tissue fragments.

**Table I:** Total number of follicles in each treatment group, at Day 0 and after 6 days of culture. **(a), (b), (c) and (d) denote a significant difference between treatment groups. A significantly greater proportion of primary and secondary follicles were observed in treatment groups compared to control (*P* < 0.05).**

Group	Non-growing follicle n (%)	Primary follicle n (%)	Secondary follicle n (%)	Total
Day-0	7029 (79.6)^a^	1681 (19.0)^a^	123 (1.4)^a^	8833
Control	1896 (29.5)^b^	4129 (64.3)^b^	401 (6.2)^b^	6426
$\begin{array}{c} 1\;\mu M\;\mathrm{bpv}\left(\mathrm{HOpic}\right)\\ 10\;\mu M\;\mathrm{bpv}\left(\mathrm{HOpic}\right) \end{array}\}6\;\mathrm{days}\;\mathrm{of}\;\mathrm{culture}$	1400 (21.4)^c^	4513 (68.9)^c^	633 (9.7)^c^	6546
	880 (11.4)^d^	6047 (78.1)^d^	818 (10.6)^c^	7745
		**Total**		**29,550**

### Assessment of follicle activation and survival

Analysis of 20 717 follicles from a total of 147 ovarian cortical tissue fragments (*n* = 15–18 fragments per group per culture) after 6 days of culture showed that the proportion of non-growing follicles declined significantly in all groups compared to D0 (Table I). This decline was balanced by a significant increase in the percentage of growing follicles (primary and secondary follicles in D0: 20.4%, control: 70.5%, 1 μM bpv(HOpic): 78.6% and 10 μM bpv(HOpic): 88.7%) (*P* < 0.001 for all groups). A greater proportion of growing follicles was observed in 10 μM bpv(HOpic) compared to control and 1 μM bpv(HOpic) (*P* < 0.001 for all groups). Secondary follicles were the most mature growing follicle stage observed in all groups. The proportion of follicles progressing to the secondary stage in the presence of bpv(HOpic) was significantly higher compared to control (1 μM: 9.7%, 10 μM: 10.6%, control: 6.2%) (*P* < 0.05). No significant difference was observed between 1 and 10 μM bpv(HOpic) (*P* = 0.530). The higher concentration of bpv(HOpic) resulted in a reduction in the proportion of morphologically healthy follicles at all stages. This was seen for both non-growing (*P* < 0.05 for all groups) and growing follicles (*P* < 0.001 for primary and secondary follicles) compared to control and 1 μM bpv(HOpic) (Fig. 1).

### The effects of bpv(HOpic) on PI3K downstream pathway activation

**Figure 2:**
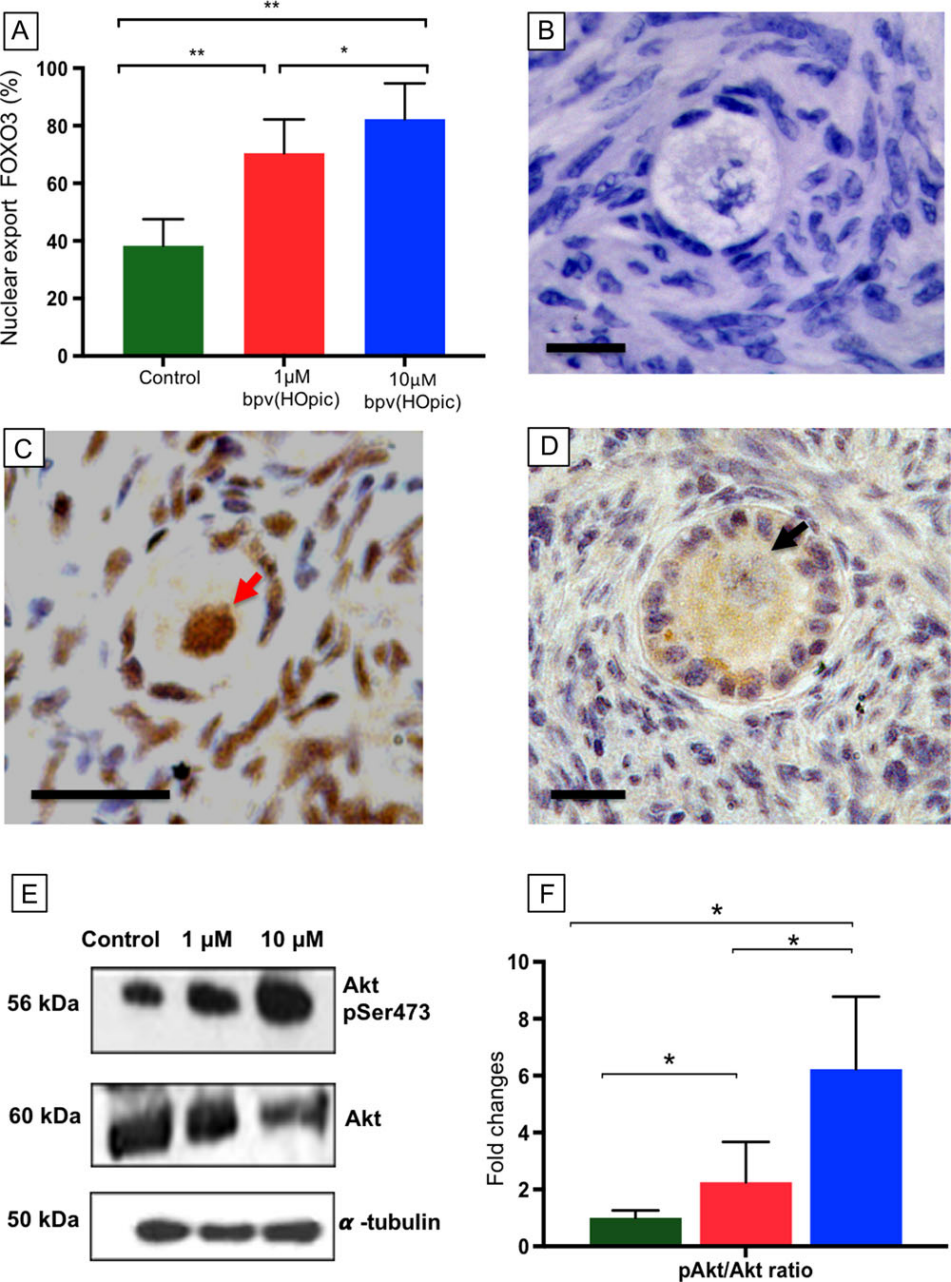
**Expression of nuclear export of FOXO3 and ratio of phosphorylated Akt and Akt in control and bpv(HOpic) treated tissue.** (**A**) Comparison of oocyte nuclear export of FOXO3 in control and bpv(HOpic) groups. Histogram shows mean percentage ± SEM (from three cultures per treatment with a minimum of 100 follicles analysed per group) of oocytes showing non-nuclear detection of FOXO3. **B**-**D**: Photomicrographs showing localisation of FOXO3 in bovine follicles. Negative control (B). Non-growing follicles with brown staining in the nucleus indicating inactivated FOXO3 (red arrow, C), nuclear export of FOXO3 from the nucleus of the activated primary follicles in bpv(HOpic) group indicated by brown staining in the ooplasm and negative staining in the nucleus (black arrow, D). Scale bar = 20 μm. (**E**) Western blot showing Akt and phosphorylated Akt (pAkt) expression in all groups. (**F**) pAkt/Akt ratio following 24 h exposure cultured control (green), 1 μM bpv(HOpic) (red), 10 μM bpv(HOpic) (blue). Lines represent significant differences between groups with a *P* value of ≤0.05 *.

To assess the nuclear exclusion of FOXO3 as an effect of PTEN inhibitor on the PI3K pathway, FOXO3 localisation was determined by immunohistochemistry (Fig. 2A–D). A total of 1704 follicles were analysed over three separate cultures and the mean percentage ± SEM of oocytes showing nuclear exclusion of FOXO3 was calculated (Fig. 2A). A significant increase in nuclear exclusion of FOXO3 was observed in oocytes contained within tissue exposed to bpv(HOpic) 1 μM (69.1 ± 11.7%; *P* = 0.008) and 10 μM (81.2 ± 12.4%; *P* = 0.003) compared to controls (38.3 ± 9.2%). Furthermore, the proportion of follicles with nuclear exclusion of FOXO3 was significantly higher in 10 μM compared to 1 μM (*P* = 0.020) (Fig. 2A).

Western blot analysis showed an increase in the ratio of pAkt (Ser473) to Akt in bpv(HOpic) exposed tissue compared to control (2.25- and 6.23-fold higher in 1 and 10 μM bpv(HOpic) respectively, *P* < 0.05) (Fig. 2E and F). A significant increase was observed in the higher concentration of bpv(HOpic) compared to 1 μM (*P* = 0.030) (Fig. 2F).

### The effects of PTEN inhibitor on DNA damage and DNA DSB repair capacity in follicles

**Figure 3:**
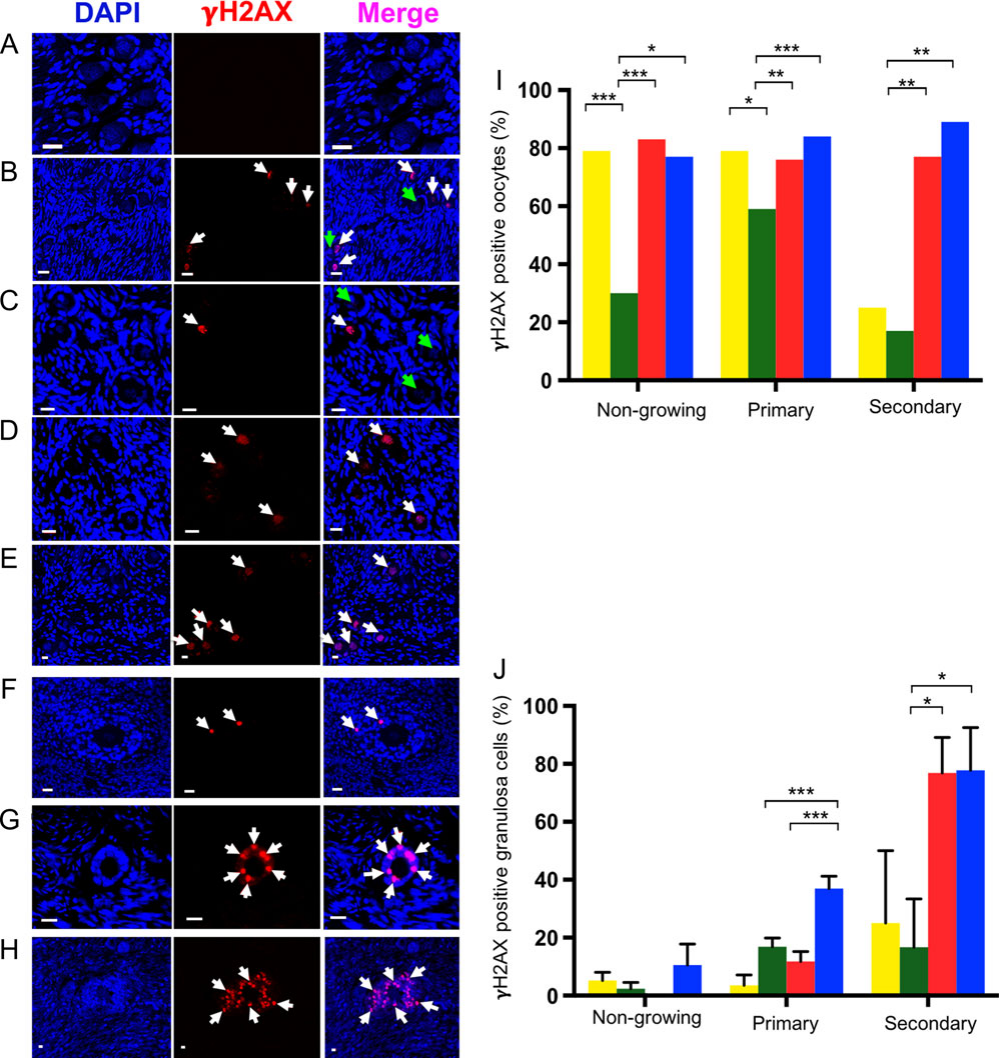
**Representative images showing localisation by immunofluorescence of γH2AX bovine ovarian tissue in each treatment group**. γH2AX (red) and DAPI (blue) staining in oocyte and granulosa cells (**A**–**H**). γH2AX staining appeared as bright points (foci) within nuclei (white arrows) in oocytes (A-E). The green arrows indicate areas where there is no γH2AX expression (B,C). Negative control (A), γH2AX positive and negative in the oocytes of day zero (D0) (B) positive and negative staining in cultured control (C); positive staining in 1 μM bpv(HOpic) (D) and 10 μM bpv(HOpic) (E). Localisation of γH2AX expression (white arrows) in granulosa cells (F–H) in control (F), 1 μM (G) and 10 μM bpv(HOpic) (H). Scale bar = 20 μm. Comparison of proportion of follicles showing γH2AX positive staining in the oocytes (**I**) and granulosa cells (**J**) of all groups. Analysis of 567 follicles from three independent experiments (I and J). The proportion of γH2AX positive oocytes per total number of follicles in each stage of follicle development (I); expression of γH2AX in granulosa cells (J), mean ± SEM. ***P ≤ 0.001, **P ≤ 0.01 and *P ≤ 0.05. Yellow, D0; green, cultured control; red,1 μM bpv(HOpic) and blue, 10 μM bpv(HOpic).

γH2AX binds at DNA strand breaks and is a marker of DNA damage. Localisation of γH2AX in each of the groups was analysed in oocytes (Fig. 3A–E) and granulosa cells (Fig. 3F–H). After 6 days of culture, γH2AX expression was reduced from 79% (D0) to 30 and 59% in non-growing and primary follicles respectively (*P* < 0.001) (Fig. 3I). Culture did not significantly affect γH2AX expression in oocytes of secondary follicles. However, bpv(HOpic) increased γH2AX expression in oocytes of all follicle types at both concentrations of bpv(HO)pic (1 μM: non-growing, 83%; primary, 76%; secondary, 77%; 10 μM: non-growing, 77%; primary, 84%; secondary, 89%) (*P* < 0.05), with no difference between doses (Fig. 3I).

In granulosa cells, γH2AX expression in non-growing follicles did not significantly differ between groups (Fig. 3J). Similarly, no significant differences were observed between D0, control and the lower concentration of bpv(HOpic) in primary follicles (Fig. 3J). A significant increase in expression in primary follicles was observed in the higher (36.9 ± 4.2) compared to the lower concentration (11.8 ± 3.39) and control (16.9 ± 2.9) (*P* ≤ 0.001) (Fig. 3J). No differences in γH2AX expression in granulosa cells of secondary follicles were observed between D0 and control; however, significant increases were observed in both bpv(HOpic) treatment groups (1 μM: 76.9 ± 12.2, *P* = 0.024; 10 μM bpv[HOpic]: 77.8 ± 14.0, *P* = 0.011) compared to control (16.7 ± 16.7) (Fig. 3F–J). There was no significant difference between the two concentrations.

**Figure 4:**
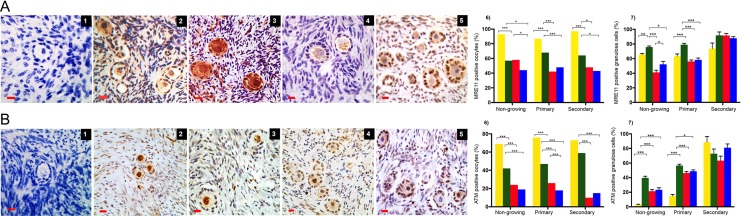
**Immunohistochemical detection of MRE11 and ATM in oocytes and granulosa cells of follicles in all groups**. Photomicrographs of MRE11 localisation (**A**1–5) and ATM (**B**1–5) expression in oocytes and granulosa cells. Negative control (1); positive staining (brown) in the oocytes and granulosa cells of day zero (D0; 2), control (3); 1 μM (4) and 10 μM bpv(HOpic) (5). Scale bar = 20 μm. The proportion of MRE11 and ATM in oocytes (A6, B6) and granulosa cells (A7, B7) shown as mean percentage ± SEM. Yellow bars, D0; green bars, cultured control; red bars, bpv(HOpic) 1 μM and blue bars, bpv(HOpic) 10 μM. Total number of follicles analysed: 4659 (MRE) and 5309 (ATM). ***≤0.001, **≤0.01, *≤0.05. *P* value was assigned at ≤0.05.

Expression of the DNA DSBs repair proteins MRE11 (Fig. 4A1–5) and ATM (Fig. 4B1–5) was observed in all stages of follicle development after 6 days of culture (Fig. 4A and B). MRE11 was decreased in oocytes in 1 μM (42%) and 10 μM bpv(HOpic) (47%) of primary follicles compared to control (68%) (*P* < 0.001) (Fig. 4 A6). Similarly, the expression of MRE11 in granulosa cells declined significantly in the presence of bpv(HOpic) in non-growing (1 μM: 41.2 ± 2.9%; 10 μM: 52.3 ± 3.9%) and primary follicles (1 μM: 56.2 ± 1.9%; 10 μM: 58.3 ± 2.5%), compared to control (non-growing: 75.9 ± 1.4% and primary follicles: 79.0 ± 1.8%) (*P* < 0.05 for all groups). No significant reduction was observed in secondary follicles with either dose (Fig. 4A7).

ATM, a regulator of the DNA repair downstream pathway, declined significantly in all types of follicles in bpv(HOpic) groups at Day 6 of culture. bpv(HOpic) reduced ATM expression in oocytes of primary follicles from 26% in 1 μM to 18% in 10 μM bpv(HOpic) (*P* ≤ 0.001) (Fig. 4B6). In granulosa cells, ATM expression in bpv(HOpic) groups of non-growing and primary follicles was significantly lower compared to control (*P* < 0.05) (Fig. 4B7).

**Figure 5:**
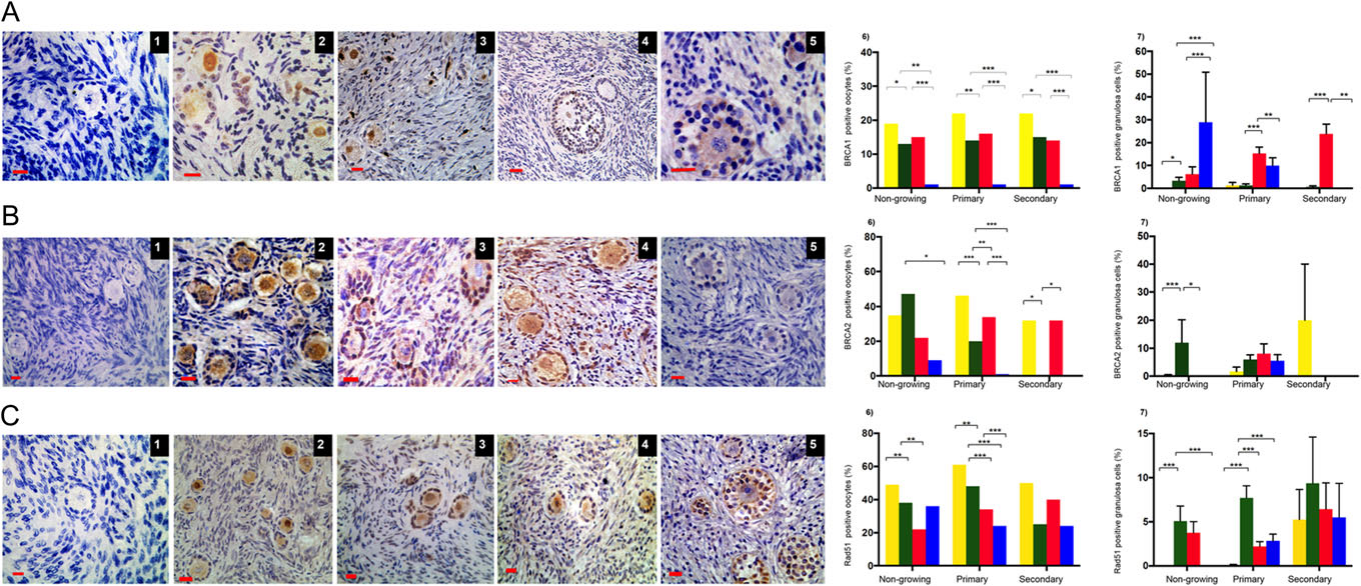
**Immunohistochemical detection of BRCA1, BRCA2 and Rad51**. Photomicrographs of BRCA1 (**A**1–5), BRCA2 (**B**1–5) and Rad51 (**C**1–5) expression in oocytes and granulosa cells. Negative control (A1, B1 and C1); positive staining (brown) in the oocytes and granulosa cells of day zero (D0; A2,B2,C2), control cultures (A3, B3 and C3); 1 μM (A4, B4 and C4) and 10 μM bpv(HOpic) (A5, B and B6). Scale bar = 20 μm. The proportion of oocytes (A6, B6 and C6) and granulosa cells (A7, B7 and C7) expressing BRCA1, BRCA2 and Rad51 in each treatment group (yellow bars, D0; green bars, cultured control; red bars, bpv(HOpic) 1 μM and blue bars, bpv(HOpic) 10 μM). Analysis of 1315 (BRCA1), 1134 (BRCA2) and 4148 (Rad51) follicles. ***≤0.001, **≤0.01, *≤0.05. *P* value was assigned at ≤0.05.

BRCA1, BRCA2 and RAD51 were localised within oocytes and granulosa cells (Fig. 5A–C). Analysis of 1315 follicles revealed that the proportion of BRCA1 positive oocytes of non-growing and primary follicles decreased significantly after 6 days of culture in control group (19 versus 13% and 22 versus 14% in non-growing and primary follicles, respectively) (*P* < 0.05) (Fig. 5A6). BRCA1 expression in all follicle types treated with 1 μM bpv(HOpic) did not change significantly compared to control (*P* > 0.05). However, there was very low expression of BRCA1 in all follicle groups treated with 10 μM bpv(HOpic) (*P* < 0.001) (Fig. 5A6). Similarly, low expression of BRCA1 was seen in granulosa cells of growing follicles treated with 10 μM bpv(HOpic) although granulosa cells of non-growing follicles showed a high level of expression (Fig. 5A7). BRCA2 expression in oocytes was markedly increased in 1 μM bpv(HOpic) in primary follicles (36%) compared to control (20%, *P* = 0.010) (Fig. 5B6). There was no significant difference in expression within granulosa cells among all the groups in primary and secondary follicles (Fig. 5B7).

In contrast, Rad51 expression in oocytes was significantly reduced in both bpv(HOpic) groups in primary follicles (control versus 1 and 10 μM bpv[HOpic]: 48 versus 34 versus 24%) (*P* < 0.05), without significant changes in secondary follicles (Fig. 5C6). Rad51 expression was observed infrequently (<10%) in granulosa cells, mainly in secondary follicles with no significant changes observed among the groups (*P* > 0.05) (Fig. 5C7).

## Discussion

Consistent with our previous finding using human tissue (McLaughlin *et al.*, 2014), bovine ovarian tissue fragments exposed to 1 and 10 μM bpv(HOpic) for 24 h showed increased primordial follicle activation. The culture system used in this study supports significant primordial follicle activation in the control group: recent studies indicate that this is as a result of disrupting the Hippo signaling pathway during the preparation of the tissue (Kawamura *et al.*, 2013; Hsueh *et al.*, 2015). Hippo disruption increases expression of downstream growth factors but manipulation of the PI3K pathway results in further activation (Kawamura *et al.*, 2013; McLaughlin *et al.*, 2014; Hsueh *et al.*, 2015; Grosbois and Demeestere, 2018). PI3K pathway activation resulting from PTEN inhibition was confirmed by increased phosphorylated Akt expression and nuclear exclusion of FOXO3. However, a deleterious effect on follicle morphological health was observed with the higher dose bpv(HOpic), in agreement with data from human ovary (Lerer-Serfaty *et al.*, 2013; McLaughlin *et al.*, 2014). The present data extend this by demonstrating that increased activation is associated with increased DNA damage and reduced DNA repair in ovarian follicles and, particularly, in oocytes.

The findings in this study support the view that the PTEN/Akt/PI3K pathway involves other intracellular pathways (Blanco-Aparicio *et al.*, 2007) that may have negative impacts on follicle growth. PTEN/PI3K/Akt activity impacts on DNA damage and repair (Hunt *et al.*, 2012; Ming and He, 2012) and has a central role in coordinating the apoptosis cascade activity (Weng *et al.*, 2001; Lu *et al.*, 2016). As DNA damage precedes the apoptotic process and can be present without any significant morphological changes, we investigated the effect of PTEN inhibition on DNA damage and DNA repair capacity of oocytes and granulosa cells. The bpv(HOpic) concentrations used in this study were low with a short-term incubation compared to other studies in human (Novella-Maestre *et al.*, 2015). However, these low concentrations clearly increased DNA damage and compromised DNA repair capacity of the follicles.

The DNA damage repair pathway involves γH2AX, and this binds specifically to the location of damage and controls recruitment of DNA repair proteins. Phosphorylation of γH2AX initiates the downstream pathway that leads to DNA repair or cell cycle arrest (Oktay *et al.*, 2015). We found that γH2AX expression was significantly higher in uncultured D0 tissue compared to control. However, the high γH2AX expression level in the D0 group was associated with increased expression of the DNA DSBs repair proteins MRE11, ATM and Rad51 at all stages of follicle development. These breaks could reflect latent damage due to mild injury during tissue preparation and transport that appear to be rapidly resolved and may not cause serious consequences. This type of damage can be repaired directly without cell cycle arrest (Menezo *et al.*, 2010), as was indicated by the reduction in γH2AX expression following tissue culture and there being fewer morphologically unhealthy follicles in the cultured control tissue. All types of follicles in control cultures generated adequate DNA repair capacity compared to treatment groups, which may reflect a culture medium with a nutrient-rich environment that is beneficial to cell metabolism (Paynter *et al.*, 1999).

DNA damage persisted or increased in the oocytes of both bpv(HOpic) treatment groups and was not associated with increasing DNA repair protein expression. In the DNA damage repair pathway, BRCA2 is indispensable in regulating the activity of Rad51. Increased BRCA2 expression in oocytes was consistent with the expression of Rad51 except in the higher dose of bpv(HOpic) of non-growing follicles wherein low level of BRCA2 did not affect Rad51 expression. In contrast, BRCA2 expression was high in 1 μM bpv(HOpic) exposed primary follicles but was not associated with increased Rad51 expression. This may indicate compromised homologous recombination. This finding may describe the association between Akt activation by bpv(HOpic) and defects in DNA damage repair protein interactions. Deficiencies in these interactions have previously been reported in human and mouse ovarian studies and associated with ageing (Titus *et al.*, 2013). DNA DSB repair capacity as reflected in BRCA1, BRCA2 and Rad51 was markedly reduced in oocytes exposed to the higher dose of bpv(HOpic). Activation of Akt has been shown to abolish the G2 cell cycle checkpoint by delaying nuclear translocation of BRCA1 during DNA DSB repair in a breast cancer cell line. This leads to deactivation of Chk1 following DNA damage process (Tonic *et al.*, 2010; Wu *et al.*, 2010). Although we did not quantitatively measure the intensity of DNA damage indicated by γH2AX expression in this study, our findings suggest that the DNA damage in the presence of bpV(HOpic) might be severe with limited repair, which may result in permanent cell cycle arrest.

Increased expression of γH2AX was observed in granulosa cells of growing follicles in the bpv(HOpic) treated groups. DNA DSB repair capacity of secondary follicles was not compromised in all groups, except BRCA1, which was apparently decreased with higher dose bpv(HOpic). Interestingly, the lower dose bpv(HOpic) did not affect expression of BRCA1 in all follicle types. Most of the follicles in higher dose bpv(HOpic) showed apoptosis after 6 days of culture. One possible explanation of these findings is that as actively dividing cells, such as granulosa cells, demonstrate a high metabolic activity and proliferation rate that will increase with the activation of Akt. In this context, granulosa cells of secondary follicles are more vulnerable to DNA induced damage. It seems likely that a decline in the capacity of DNA repair in granulosa cells happens more slowly than DNA damage, similar to the process that occurs with ageing (Zhang *et al.*, 2015). The proportion of morphologically normal follicles did not vary between 1 μM bpv(HOpic) and control group regardless of the presence of DNA damage and lack of DNA repair capacity. This may reflect a better response to DNA damage in low-dose compared to high-dose group but a study on human tissue has shown that the growth of apparently healthy preantral follicles isolated after treatment with 1 μM bpv(HOpic) was compromised after a further six days of culture (McLaughlin *et al.*, 2014). It has been reported that different factors affect the time period between the occurrence of DNA damage and apoptotic events (Xiao *et al.*, 2017). This study indicates that the dose of bpv(HOpic) could also affect this time frame.

It is worth considering the broader significance of these findings since PTEN inhibition has been used to activate primordial follicles in POI patients by activating follicles in tissue that is subsequently grafted back to patients (Suzuki *et al.*, 2015). The present data suggest that this strategy may be associated with increased DNA breaks and reduced DNA repair capacity. The impact of DNA damage on oocytes may range from meiotic dysfunction to cell death (Oktay *et al.*, 2015), possibly leading to reduced fertility (Kirk and Lyon, 1982; Meirow *et al.*, 2001; Menezo *et al.*, 2007; Adriaens *et al.*, 2009). More than 50% of oocytes with severe DNA DSBs can escape apoptosis and eventually achieve resumption of meiosis to the germinal vesicle breakdown stage in mice, but none of these oocytes develop to metaphase II (Lin *et al.*, 2014). This indicates that intact DNA DSB repair capacity in oocytes is pivotal to achieving mature and competent oocytes capable of fertilisation. This study is limited to primordial follicle activation and implications for later stages of follicle development have not been assessed. Impairment of human preantral follicle growth has been demonstrated after bpv(HOpic) treatment *in vitro* (McLaughlin *et al.*, 2014), but the implications for mature oocyte development are unexplored. We have recently demonstrated that a human *in vitro* growth (IVG) system (whose first stage is as used here) can support complete follicle development resulting in metaphase II oocytes (McLaughlin *et al.*, 2018). This methodology may be useful to provide additional insights into DNA damage and DNA repair of oocytes and granulosa cells, which may subsequently lead to improved IVG systems.

In summary, this study demonstrates that increasing activity of the PI3AKT pathway by a short exposure of bovine ovarian tissue fragments to bpV(HOpic) results in increased primordial follicle activation. However, this was accompanied by increased DNA damage and compromised DNA DSB repair capacity, in both oocytes and granulosa cells. These findings highlight the complexities and interactions between the regulation of initiation of follicle growth and the maintenance of follicle health and indicate the need for caution in developing pharmacological approaches to manipulation of this pathway for clinical use.
